# Association between parental feeding practices and children’s dietary intake: a cross-sectional study in the Gardermoen Region, Norway

**DOI:** 10.29219/fnr.v66.8050

**Published:** 2022-03-21

**Authors:** Marlene Mazza, Marianne Morseth, Liv Elin Torheim

**Affiliations:** Department of Nursing and Health Promotion, Faculty of Health Sciences, Oslo Metropolitan University, Oslo, Norway

**Keywords:** parental feeding practices, food intake, K diet, preschoolers

## Abstract

**Background:**

Parental feeding practices may be important determinants for children’s diets. In Norway, few studies have assessed this association and to our knowledge, no studies have included fish as an outcome.

**Objective:**

The purpose of this study was to explore the association between multiple parental feeding practices and children’s food intake.

**Design:**

Parents (n = 111) of preschool children aged 1–5 years in the Gardermoen Region in Norway were recruited. The parents completed a web–based questionnaire regarding the use of 12 feeding practices measured by the Comprehensive Feeding Practices Questionnaire (CFPQ). Children’s weekly food intake was measured using a food frequency questionnaire (FFQ). The association between parental feeding practices and food intake was assessed by logistic regression.

**Results:**

The feeding practices involvement and environment increased the likelihood of children having a higher intake of fruit and berries (OR = 1.99, 95% CI = 1.15, 3.44 and OR = 2.10, 95% CI = 1.17, 3.78, respectively) when controlling for potential confounders. A positive association was found between the feeding practice environment and the children’s intake of vegetables (OR = 2.94, CI = 1.55, 5.55), and between modeling and intake of vegetables (OR = 2.14, CI = 1.26, 3.63). Also, the feeding practice encourage balance and variety increased the likelihood of a higher consumption of vegetables (OR = 5.18, CI = 1.63, 16.5). Parents who more frequently encouraged the child to eat balanced and varied were more likely to have children with a higher consumption of fish (OR = 5.03, CI = 1.62, 15.7). If parents used more restriction for weight, the child was less likely to have a high SSB consumption (OR = 0.43, CI = 0.22, 0.83).

**Conclusion:**

Findings suggest that children’s intake of the favorite food item groups, fruit and berries, vegetables and fish, was associated with the use of positive feeding practices, such as involvement, environment, modeling and encouragement. For unfavorable food groups, only restriction for weight was negatively associated with SSB consumption. Findings should be interpreted carefully due to the relatively small sample size.

## Popular scientific summary

- Parental feeding practices may be important determinants for children’s diet and health.
- In this study, the feeding practices modeling, involvement, environment, encouragement, and restriction for weight were associated with a favorable food intake.
- Interventions promoting these practices may lead to healthier diets in children.

A healthy diet is essential for healthy growth and development in children (1). As food behaviors acquired in early life may persist into adulthood, poor dietary habits at an early age may have a great impact on both short- and long-term health outcomes (2, 3). According to national surveys, Norwegian children in general have an inadequate consumption of fruits, vegetables, and fish. Also, the children’s diets consist of too much saturated fatty acids and added sugars (4, 5). Importantly, parents serve as gatekeepers for young children’s food intake and preferences (2). Studies suggest parents’ use of different feeding practices may affect child outcomes such as food preferences, food intake and consequently weight (6–10).

Feeding practices represent a parent–child interaction, involving specific behaviors aiming to influence children’s dietary intake (11, 12), including what, how, and when children are fed (13). These practices may be context-specific and vary over time and among different children (7). Primarily, the feeding practices pressure, restrictions, and monitoring are most studied in relation to children’s food intake and are shown to have a negative impact on children’s healthy eating (11). The use of pressure to eat has in previous studies been associated with increased daily consumption of sweets and snack (14–16), and a lower intake of fruit and vegetables (14, 17, 18). Similarly, less use of pressure to eat is shown to be associated with higher consumption of fruit and vegetable (2, 15). Role modeling is found to be related to higher intake of vegetables (2) and fruits (19). Further, another study points to using food as a reward as a significant predictor of low fruit and vegetable intake, and parents allowing a child more control as a predictor for increased fruit and vegetable intake (16).

Previous studies that examine parental feeding practices in relation to children’s food intake have mostly focused on intake of vegetables, fruits, and, to some extent, sugar sweetened beverages (SSB). Little is known about the association between parental feeding practices and intake of sugars from foods, and no identified studies included intake of fish.

There are several papers on feeding practices in Norway. However, the studies are limited to toddlers <1 years, and to the intake of fruits and vegetables (20–22). Moreover, some studies conducted in Norway have explored feeding practices in relation to other endpoints than food intake, like shared family meals (21, 22). The main aim of this study was to explore the possible association between parental feeding practices and the intake of vegetables, fruits/berries, fish, foods with added sugars, and SSB in Norwegian preschoolers.

## Methods

### Sample and recruitment

The sample of the study comprised parents (>18 years) of children aged 1–6 years, attending kindergarten in the Gardermoen Region, Norway. Only one parent was invited to answer, and fathers were encouraged to participate. The parent was informed to answer for one child only. Further, if parents had more than one child in the relevant age group attending kindergarten, they were asked to answer for the oldest child to minimize bias. The parents were recruited through kindergartens. As most Norwegian children attend kindergarten (23), this recruitment method allowed many parents to be reached effectively. Also, the kindergarten staff obtain the parent’s contact information and may easily reach out to the parents. The kindergartens and parents of preschoolers were recruited through e-mail in autumn 2019. Each of the 19 kindergartens in a municipality in the Gardermoen Region was invited. First, an invitation containing information about the study to participate was sent out to kindergartens. Then, kindergartens who agreed to participate sent out an e-mail with the questionnaire to the parents in their respective kindergartens. Reminders to participate were sent out to both kindergartens and parents by e-mail. To increase participation rate, non-respondents were contacted again at least once (24). To encourage study participation, we emphasized anonymity and easy access to the web questionnaire. In total seven of the 19 kindergartens agreed to participate in the study. Three parents were excluded from the study either because of not meeting the age criteria or because of restrictive food avoidance in the child’s diet. Of a population of 473 parents in the participating kindergartens, 111 parents (23%) were included in this study.

### Measures

Parents answered a self-administered web-based questionnaire consisting of the three parts: 1) background information about the parent and the child, 2) parental feeding practices measured by a translated and validated version of the ‘Comprehensive feeding practices questionnaire’ (CFPQ), and 3) a food frequency questionnaire (FFQ) measuring the children’s weekly intake of five different food groups (vegetables, fruits and berries, fish, food with added sugars, and SSB). For background variables, the parents reported their age, gender, educational level, income level of the household, as well as the child’s age and gender. Concerning exclusion criteria, parents were also asked to report any child food avoidance. All responses were categorized to ensure anonymous data.

Feeding practices were measured using the CFPQ developed by Musher-Eizenman et al. (25). The tool was originally developed to measure multiple feeding practices among parents of 2–8 years old children. The original questionnaire includes 49 items covering 12 subscales of feeding practices (Monitoring, Emotion Regulation, Food as Reward, Child Control, Modeling, Restriction for Weight, Restriction for Health, Teaching Nutrition, Encourage Balance and Variety, Pressure to Eat, Healthy Food Environment, and Involvement). The Norwegian validated version by Melby et al., which was slightly modified and validated for parents of 10–12 years old children, consists of 45 items measuring the 12 feeding practices (26). This questionnaire has later been used in a study of 1-year-old children (27) and was deemed suitable for our study. Two different five-–point response scales were used, depending on whether the items addressed frequency or degree of compliance. The response categories were as follows: ‘never,’ ‘rarely,’ ‘sometimes,’ ‘mostly,’ and ‘always’ or ‘disagree,’ ‘slightly disagree,’ ‘neutral,’ ‘slightly agree,’ and ‘agree’ (25).

The authors adapted the FFQ from two previous national studies among Norwegian toddlers and preschoolers (4, 5). Weekly intake of five different food groups was measured: fruits/berries (cooked, fresh, canned, or frozen), vegetables (raw, e.g. salad or cooked, e.g. soup), fish (fish fillet fat or lean, e.g. salmon, or fish products), SSB (juice, nectar, soda, or other soft drinks with added sugars), and foods high in sugar (sweet snacks such as candy, chocolate, ice cream, cakes and cookies, as well as sweet toppings and cereals). A response scale with seven categories was used to capture the frequency of intake with the following response alternatives and scores: ‘seldom/less than 1 time a week’ (score: 0.5), ‘1–3 times a week’ (score: 2), ‘4–6 times a week’ (score: 5), ‘once daily’ (score: 7), ‘twice or more per day’ (score: 14), and ‘three times or more every day’ (score: 21) (28, 29).

### Statistical analysis

Based on scores from every item from the feeding practice subscales, a mean score was calculated for the 12 feeding practices. For children’s food intake, the frequencies were summarized to calculate the weekly intake for each of the five food groups. Because of the ordinal level of intake data and for better interpretation of results, they were dichotomized into low and high intake based on the median value. Descriptive statistics are presented with *n* and percentage (%) (Table 1). Associations between parental feeding practices as continuous independent variables and children’s food intake as dichotomous dependent variables were assessed using binary logistic regression. The analysis was run separately in crude and adjusted models. The models were adjusted for child age, parental age, and educational level. Based on the large number of regression analyses performed, a significance level of *P* < 0.01 was applied to limit the risk of significant results occurring by chance. A restrictive approach with adjusted *P*-values was decided for the analysis. Although *P*-value adjustments such as the Bonferroni correction may not be required when conducting exploratory analysis, multiple regression models, such as in our study, increase the likelihood of significant results occurring by chance (30). Data were analyzed using IBM SPSS® statistics, versions 25.0 and 27.0 (SPSS Inc, Chicago, IL, USA).

**Table 1:** Sample characteristics (*n* (%))

Sample characteristics	*n*	%
**Total**	**111**	**100**
**Parent**		
Mother	91	82
Father	20	18
**Age**		
18–29 years	14	13
30–34 years	46	41
35–39 years	36	32
≥40 years	15	14
**Education level**		
High school or less	25	23
Vocational education	20	18
Higher education (1–4 years)	46	41
Higher education (>4 years)	20	18
**Yearly household income**		
0–599,000 NOK	27	24
600,000–749,000 NOK	14	13
750,000–999,000 NOK	39	35
≥1,000,000 NOK	31	28
**Child’s gender**		
Female	58	52
Male	52	48
**Child’s age**		
1 year	10	9
2–3 years	47	42
4–5 years	54	49
**Food avoidance^a^**		
Yes	21	19
No	90	81
**Child from earlier** ^b^		
Yes	58	53
No	52	47

a The parent avoided feeding specific foods or drinks to their child.

b If the child has older siblings.

## Results

### Study sample characteristics

Sample characteristics are presented in Table 1. Of the 111 parents who responded, the majority (82%) were the child’s mother. The median yearly household income was 750,000–999,000 NOK. Most of the parents (59%) had an academic education. Among the children in this study, 52% were girls, and 48% were boys. The majority of children (91%) were between 2 and 5 years of age, where 42% were in the age group of 2–3 years, and 49% were in the age group of 4–5 years. One-year-old children contributed only to 9% of the sample.

### Feeding practices

The median (interquartile range [IQR]) frequency of use of the parental feeding practices is presented in Table 2. Encourage balance and variety was the feeding practice most frequently used (mean = 4.6, standard deviation [SD] = 0.4), followed by modeling (mean = 4.4, SD = 0.9) and monitoring (mean = 4.2, SD = 0.4). In contrast, parents reported less frequent use of food as a reward (mean = 1.6, SD = 0.8), emotional regulation (mean = 1.8, SD = 0.9), and restriction for weight (mean = 1.9, SD = 0.7).

**Table 2:** Frequency of use of parental feeding practices and weekly dietary intake among children (Gardermoen Region, Norway [2019], *n* = 111)

Feeding practice	Mean (SD)
Monitoring	4.2 (0.4)
Encourage balance and variety	4.6 (0.4)
Pressure to eat	2.6 (1.0)
Healthy food environment	4.0 (0.7)
Food as reward	1.6 (0.8)
Modeling	4.4 (0.9)
Involvement	3.9 (0.8)
Child control	2.6 (0.6)
Teaching about nutrition	3.9 (0.8)
Emotional regulation	1.8 (0.9)
Restriction for health	2.8 (0.9)
Restriction for weight	1.9 (0.7)
**Dietary intake**	**Median (IQR)**
Fruits and berries	6 (4–8)
Vegetables	7 (4–12)
Fish	9 (7–16)
SSB	2 (0–2)
Foods with added sugars	6 (4–7)

### Food intake

The children’s food intake is presented in Table 2. Median (IQR) intake of fruits and berries was 6 (4–8) times a week. The weekly median (IQR) intake of vegetables was 7 (4–12) per week. Parents reported that the children consumed foods high in added sugars more frequently than they consumed SSB. The median (IQR) consumption of fish among the included children was 9 (7–16) times a week.

### Associations between parental feeding practices and child food intake

Five of the feeding practices were significantly associated with food intake (Table 3). Use of the feeding practice involvement was associated with a more frequent consumption of fruits and berries (odds ratio [OR] = 1.99, 95% confidence interval [CI] = 1.15, 3.44). The feeding practice ‘healthy food environment’ was positively associated with child intake of both fruits and berries (OR = 2.1, 95% CI = 1.17, 3.78) and vegetables (OR = 2.94, 95% CI = 1.55, 4.44). A positive association was also found between the use of modeling and intake of vegetables (OR = 2.14, 95% CI = 1.26, 3.63). Parents using encouragement more frequently had children with a higher intake of both vegetables (OR = 5.8, 95% CI = 1.63, 16.5) and fish (OR = 5.03, 95% CI = 1.6, 15.7) than children whose parents did not use these feeding practices frequently. A significant association was found between restriction for weight and SSB, where children of parents using restriction for weight had lower odds of consuming SSB (OR = 0.43, 95% CI = 0.22, 0.83).

**Table 3:** Association between parental feeding practices and child food intake, Gardermoen Region, Norway (2019) (*n* = 111)

Feeding practice	Fruits/berries^a^		Vegetables^a^		Fish^a^		SSB^a^		High-sugar Foods^a^	
	OR^b^ (95% CI)	*P*	OR^b^ (95% CI)	*P*	OR^b^ (95% CI)	*P*	OR^b^ (95% CI)	*P*	OR^b^ (95% CI)	*P*
Involvement	1.99 (1.15, 3.44)	**0.01**	1.25 (0.73, 2.16)	0.42	0.89 (0.53, 1.49)	0.67	1.58 (0.92, 2.72)	0.10	0.86 (0.51, 1.45)	0.57
Environment	2.10 (1.17, 3.78)	**0.01**	2.94 (1.55, 5.55)	**<0.01**	1.05 (0.61, 1.82)	0.86	0.55 (0.29, 1.03)	0.06	0.54 (0.29, 0.98)	0.04
Emotional regulation	0.77 (0.49, 1.21)	0.26	0.75 (0.47, 1.19)	0.23	0.92 (0.59, 1.44)	0.71	0.80 (0.49, 1.31)	0.37	1.25 (0.78, 1.99)	0.35
Food as reward	1.12 (0.67, 1.87)	0.66	0.95 (0.56, 1.60)	0.84	1.05 (0.64, 1.75)	0.84	0.90 (0.53, 1.53)	0.70	0.98 (0.58, 1.64)	0.94
Pressure	0.78 (0.52, 1.17)	0.23	0.94 (0.62, 1.43)	0.76	0.93 (0.62, 1.39)	0.72	1.21 (0.79, 1.85)	0.39	1.27 (0.84, 1.92)	0.27
Modeling	0.95 (0.60, 1.50)	0.83	2.14 (1.26, 3.63)	**<0.01**	1.54 (0.95, 2.48)	0.08	0.97 (0.60, 1.57)	0.90	0.88 (0.56, 1.41)	0.60
Monitoring	1.29 (0.64, 2.62)	0.48	0.78 (0.37, 1.67)	0.52	1.42 (0.70, 2.89)	0.33	1.32 (0.64, 2.72)	0.45	0.45 (0.20, 1.02)	0.05
Encouragement	1.87 (0.66, 5.29)	0.24	5.18 (1.63, 16,5)	**<0.01**	5.03 (1.62, 15.7)	**<0.01**	1 (0.33, 3.03)	1.00	0.71 (0.25, 2.06)	0.53
Child control	0.87 (0.45, 1.70)	0.68	0.56 (0.28, 1.12)	0.10	0.73 (0.38, 1.42)	0.36	0.69 (0.33, 1.44)	0.32	1.74 (0.87, 3.49)	0.12
Teaching nutrition	1.79 (1.06, 3.03)	0.03	1.98 (1.14, 3.45)	0.02	1.41 (0.85, 2.34)	0.19	0.71 (0.42, 1.22)	0.22	0.88 (0.53, 1.46)	0.62
Restriction health	0.77 (0.50, 1.17)	0.22	0.95 (0.62, 1.47)	0.82	0.93 (0.62, 1.40)	0.73	0.69 (0.44, 1.08)	0.11	0.91 (0.60, 1.39)	0.67
Restriction weight	1.48 (0.81, 2.71)	0.20	1.33 (0.71, 2.49)	0.37	1.59 (0.88, 2.89)	0.39	0.43 (0.22, 0.83)	**<0.01**	0.64 (0.35, 1.19)	0.54

a Divided into low or high intake based on median value. Low intake is the reference group.

b Adjusted for child’s age, and educational level and age of parent. Significant *P-*values (*P* < 0.01) in bold.

## Discussion

In this sample of parents of preschoolers in the Gardermoen Region in Norway, the five feeding practices – modeling, encouragement, environment, involvement, and restriction for weight – were found to be significantly associated with child food intake.

We found that the modeling practice (parent models healthy eating) was positively associated with vegetable consumption, which is in line with other studies (2, 31, 32). One explanation may be that parents who model the consumption of healthy foods consume these foods themselves, making them more available for children to eat (32). Also, by modeling consumption of healthy foods, the parents may teach children about healthy eating (33). In line with this, Quah et al. found that modeling was associated with a lower consumption of unhealthy foods, such as sweets and snacks (32).

The encouragement practice measuring parent’s encouragement to consume a balanced and varied diet was positively associated with consumption of both fish and vegetables. However, wide CIs for these estimates may indicate less precision in the estimated OR, leaving this association less certain. The feeding practice encouragement has previously been shown to be associated with a favorable diet in children including a higher vegetable intake (32–34) and higher intake of fruits (35). In a study conducted among Norwegian children with an average age of 10.9 months, encouraging balance and variety was a significant mediator on the children’s vegetables intake (20). Findings thus indicate that parents encouraging greater balance and variety may lead to healthier food consumption in children.

Healthy food environment (parents making healthy foods available in the home food environment and unhealthy foods less accessible) was positively associated with children’s intake of fruit/berries and vegetables. In the above-mentioned study by Røed et al., researchers found a relationship between a healthy food environment and a higher intake of both fruit and vegetables. Their findings showed that a healthy food environment was a significant mediator in the association between parents’ health motives and children’s intake of fruit and vegetables (20). Melbye and Hansen (36) reached a conclusion similar to ours, in a study conducted among older children (aged 10–12 years) in Norway. This may indicate that the association between child food intake and a healthy food environment is consistent both in childhood and young adolescence.

In our study, involvement was another feeding practice positively associated with children’s fruit intake. Results from other studies also show an association between parental involvement and children’s food intake. However, associations were found with vegetable intake only (33, 35).

Further, restriction for weight (parents restricting foods with the motive to control the child’s weight) was negatively associated with consumption of SSB in children, thereby the only practice associated with one of the two unfavorable food groups. This contradicts findings from another study where no association between parental use of restriction for weight and child SSB consumption was found (32). Some researchers point out that restrictions may be beneficial for children’s healthy diets at an early age (37–39). However, when the child is older, restriction of food may increase the preference for and consumption of that specific food (40, 41). This leads to questions regarding the direct impact of feeding practice on children’s diet versus the long-term effect on child food preferences and food consumption.

In this study, all five feeding practices associated with child food intake were related to a favorable food intake only. No practice was found to promote an unhealthy diet (measured by high SSB consumption or sugary foods, or a low intake of fruits, vegetables, or fish), which contradicts previous findings (2, 14, 15, 17, 18, 42, 43). This may be explained by different tools used to capture restrictive feeding practices, but also by the difference in variables included in the analysis causing mixed results (44). Finally, one explanation may be the complexity of measuring human interaction and behaviors, such as feeding practice (7).

The main strengths of our study include highlighting this association in a Norwegian context, additionally including fish as an intake variable. Overall, this study increases the level of understanding in this study field. Given that data on both parental feeding practices and dietary intake were collected concurrently, the dietary intake responses are most likely not affected by parent’s awareness of their feeding practices. Our analysis distinguished between the fruit and vegetable categories, whereas most studies tend to combine these into one group (33). This is of importance in our study because the children’s vegetable intake was lower than the fruit intake, and the association between feeding practice and consumption of these two food groups may differ. As Norwegian children in general consume twice as much fruits as vegetables (5), it is important to investigate whether certain feeding practices may have a positive influence on children’s vegetable intake. Mothers, argued to be primary caregivers, are often the main focus in research regarding children’s diets (45). Given that fathers’ exert influence on their children’s eating behaviors, fathers’ food parenting warrants attention (7). However, fathers’ participation in this study turned out low.

However, there are limitations to our study that should be considered. The main limitation is the low response rate. The small sample (*n* = 111) concerns whether the findings can be generalized to the respective population. A low response rate may increase the likelihood of selection bias (24). Web-based surveys tend to generate a low response rate, and the comprehensive and long questionnaire may have generated dropouts (24).

Also the representation of different subgroups of the population may potentially contribute to limiting the generalizability of these findings. In this study, men, although encouraged to participate, were underrepresented. The 1-year-old children contributed to only 9% of the sample. In addition, the municipality has a relatively low socioeconomic status (46), while the study sample in general had a high academic educational level. Therefore, the study results probably may be generalized to the part of the population with higher SES only.

Although a web-based questionnaire and self-reporting of data may be favorable to increase participation, the possibility of misreporting is present. Parents may answer what is socially desirable, which may lead to systematically biased results. However, using a tool that has been externally validated and respondent anonymity increase the likelihood of valid reports. The cross-sectional design was suitable for the purpose of this study, timeframe, and available resources considered. However, it is important to point out that this design does not allow for casual inferences, and there is a possibility of reverse causation (32). It cannot be determined whether child food intake is caused by the use of a specific practice, or if the use of parental feeding practices is influenced by, and adapted to, the child.

## Conclusion

In order to improve child diet and health, understanding how parents as key players affect their children’s food intake and preferences is of importance. This study indicates that some parental feeding practices may be related to children’s healthy eating. Both the feeding practices involvement and healthy food environment were positively associated with higher consumption of fruits and berries. The three practices – healthy food environment, modeling, and encouragement – were all positively associated with vegetable intake. Parental use of these feeding practices may thus lead to a favorable diet in children. Parents using more restrictions for weight had children with lower consumption of SSB. This understanding may help to inform the development of more tailored interventions aiming to promote healthy diet in children.
